# Temperament and character in an Australian sample: examining cross-sectional associations of personality with age, sex, and satisfaction with life

**DOI:** 10.7717/peerj.15342

**Published:** 2023-05-11

**Authors:** Diann S. Eley, Vikas Bansal, C. Robert Cloninger, Janni Leung

**Affiliations:** 1 Academy for Medical Education, Medical School, The University of Queensland, Brisbane, Queensland, Australia; 2 Medical School, The University of Queensland, Brisbane, Queensland, Australia; 3 Anthropedia Foundation, St Louis, MO, USA; 4 Faculty of Health and Behavioural Sciences, The University of Queensland, Brisbane, Queensland, Australia

**Keywords:** Personality, Temperament, Character, Well-being, Satisfaction with life, Affect, Cross-cultural comparisions, Australian general population, Public health

## Abstract

**Objective:**

Personality can influence how we interpret and react to our day-to-day life circumstances. Temperament and character are the primary dimensions of personality, and both are influenced genetically. Temperament represents our emotional core, while character reflects our goals and values as we develop through life. Research shows that where people live, their social, economic, and physical environment can influence attitudes and behaviors, and these have links to variations in personality traits. There are few studies that focus on Australian personality as temperament and character. Using an Australian general population sample, we examined the psychometric properties of the Temperament and Character Inventory (TCIR140) and investigated the associations between TCIR140 traits with both sociodemographic variables and measures of well-being. In addition, we investigated differences in temperament and character between our Australian general population sample and published results of similar studies from other countries.

**Methods:**

Australians (*N* = 1,510) completed the Temperament and Character Inventory (TCIR-140), the Positive and Negative Affect Scale and the Satisfaction with Life Scale. Cronbach’s alpha and confirmatory factor analysis (CFA) examined the TCIR-140 psychometrics. Correlation analyzes, independent sample *t*-tests and ANOVA with post-hoc comparisons analyzed the sample.

**Results:**

Cronbach’s alphas were high, ranging from *α* = 0.78–0.92, and the CFA confirmed two constructs of temperament and character. Females were higher in Harm Avoidance (*p* < 0.001), Reward Dependence (*p* < 0.001), and Cooperativeness (*p* < 0.001) compared to males, who were higher in Self-Directedness (*p* < 0.001). Age groups showed significant differences among all temperament and character traits (*p* < 0.001) except for Reward Dependence (*p* = 0.690). Young adults had the least resilient personality profile and poorest measures of well-being. Correlations with measures of temperament and character, well-being and affect were all in the expected direction.

**Conclusions:**

Temperament and character are related to indicators of wellbeing and differs by age and sex. This Australian sample demonstrate a temperament that is high in Persistence and a character high in Self-Directedness and Cooperativeness with an overall postive affect and a general satisfaction with life. In comparison to other countries, Australians in this sample differ in levels of several traits, demonstrating a cautious and independent temperament with a character that is cooperative, industrious, and self-reliant. Young-adults in comparison to older groups have a temperament and character profile that is prone to negative emotions and a lower satisfaction with life.

## Introduction

Understanding personality continues to be an important part of how individuals think about and interpret their surroundings and interactions with others. Since the 1980s, personality psychology has grown to include constructs such as values, emotion, identity, and well-being (Roberts et al., 2007). Well-being is strongly associated with personality. Although personality in general is often discussed in terms of stereotype, (*e.g.*, introvert or extrovert) every individual is distinguished by a unique combination of traits (Cloninger & Zwir, 2018). In terms of subjective well-being, personality can have a stronger influence than life circumstances because temperament and character contribute a heritable component of personality (Cloninger & Zohar, 2011; Diener, 2009). This does not suggest that life circumstances have no influence on our feelings or behavior, or that over time, our personality will not change. On the other hand, subjective well-being, like our personality is relatively stable over time.

There is a substantial literature that has studied this association across countries and cultures. However, with few exceptions, the Five Factor Model of personality is used (McCrae & Allik, 2002). Because of its distinction between non-intentional (temperament) and intentional (character) domains of personality (Cervone, 2005), which is not addressed by other personality models, this study used the Biopsychosocial Model of Personality, which is operationalized by the Temperament and Character Inventory (TCI) (Cloninger, 1994; Cloninger, Svrakic & Svrakic, 1997).

The TCI measures the seven basic traits of personality through the dimensions of temperament and character. Temperament represents our emotional core and reflects responses to external and internal stimuli, whereas character reflects self-concepts that influence an individual’s goals and values. Although temperament develops in childhood and tends to remain steady throughout life, both dimensions are shown to have strong heritability (Gillespie et al., 2003; Zwir et al., 2020a; Zwir et al., 2020b).

The four temperament traits are Novelty Seeking (NS), Harm Avoidance (HA), Reward Dependence (RD), and Persistence (PS). Novelty Seeking measures an emotional drive to activate behavior because of curiosity to explore and to enjoy what is new and complex. Harm Avoidance measures an emotional drive to inhibit behaviour because of a tendency toward anxiety and worry that anticipates problems and failure. Reward Dependence measures an emotional drive that is characterized by behavior that pursues signals of social approval and attachment. Persistence measures an emotional drive for achievement through indefatigable ambition and determination (Josefsson et al., 2011; Josefsson et al., 2013).

The three character traits are Self-Directedness (SD), Cooperativeness (CO), and Self-transcendence (ST). Self-Directedness measures intra-personal character strengths, such as being responsible, purposeful, and admitting one’s own faults realistically. It has been shown to be the strongest overall predictor of well-being (Cloninger & Zohar, 2011; Josefsson et al., 2011). Cooperativeness measures character strengths toward emotional maturity and a strong personal identity typified by working with others for mutual benefit. Self-transcendence relates to a person’s strength of identity which is external to the self and encompasses how they believe they fit in the world around them. Character traits describe an individual’s beliefs about and attitudes toward others, life, and the universe. Therefore, character is also the domain of personality that changes more rapidly in response to personal effort, social influences, and education (Josefsson et al., 2013).

### TCI and other contemporary personality measures

Although the study of personality originated in the 1800’s, contemporary personality trait theories could arguably have stemmed from the work of Hans and Sybil Eysenck in 1975 (1916–1997). Eysenck proposed a two-axis measure of personality using extraversion-introversion and neuroticism-emotional stability. He later added psychoticism and hence developed the PEN model of personality, *i.e.,* psychoticism, extraversion, and neuroticism (Eysenck, Eysenck & Barrett, 1985). Germaine to this article, and controversial at the time, is that he believed that biological factors, in particular the hereditary influence of genes played a vital role in one’s behavior.

The most recognized today are models that analyze differences in terms individual attitudes and behaviors across broad measures of personality. The Five-Factor personality model (FFM) (Costa & McRae, 1992) is a popular measure. The FFM was derived empirically from factor analysis to describe five main measures of personality, which are, Openness to Experience, Conscientiousness, Extraversion, Agreeableness and Neuroticism. There are other models measuring more dimensions such as the HEXACO, which although outwardly similar to the FFM includes three substantially different dimensions (Lee & Ashton, 2004). Another example is Cattell’s 16 PF (16 Personality Factors Questionnaire) comprising 16 distinct dimensions, clearly in disagreement with Eysenck’s theory of personality measurement (Cattell, Cattell & Cattell, 1995). Congruent with Eysenck’s model, the TCI was designed to measure the dynamic, non-linear configuration of personality by taking into account the complex architecture of the psychobiological mechanisms that contribute to individual adaptive processes

There is substantial overlap statistically in the content of the TCI and other popular personality inventories. The TCI accounts for all components of alternative tests of personality, such as the Five-Factor Model, and predicts clinical indicators of mental health as well as or better than other personality inventories (Grucza & Goldberg, 2007). There are significant correlations between the TCI and the NEO-PIR, for example, Harm Avoidance is correlated with Neuroticism (*r*2 = 0.55, *p* < 0.01), and Extraversion (*r*2 =  − 0.63, *p* < 0.01). Self-Directedness and Persistence are both correlated with Conscientiousness (*r*2 = 0.51, *p* < 0.01), (*r*2 = 0.50, *p* < 0.01) respectively. Cooperativeness and Agreeableness are positively related (*r*2 = 0.64, *p* < 0.01) (Cloninger, Svrakic & Svrakic, 1997; Cloninger, 2006; Grucza & Goldberg, 2007).

Cloninger’s psychobiological model of personality stresses the contributory interaction of biological mechanisms and learning within the context of one’s environment. Within an iterative epigenic process, the dimensions are distinct, yet each interacts with the other in influencing behavior (Cloninger, 2003). The TCI was designed to measure the dynamic structure of personality and therefore accounts for normal and abnormal personality, including mature, healthy patterns and personality disorders and a wide range of related psychopathology (Cloninger, 2006). For example, research has demonstrated that the TCI traits show relationships between perceptions of well-being, and happiness (Cloninger & Zohar, 2011; Josefsson et al., 2011) and resilience (Eley et al., 2013; Eley et al., 2013; Eley et al., 2017). The personality profile showing a temperament that is low in Harm Avoidance and high in Reward Dependence, and a character that is high in Self-Directedness and Cooperativeness is consistently associated with well-being. Self-Transcendence is also associated with well-being when combined with high Self-Directedness and Cooperativeness but can also increase the risk of ill-being under some circumstances (Josefsson et al., 2011).

### The TCI and demographic characteristics

A consistent finding regarding sex differences is that females are higher in levels of Harm Avoidance, Reward Dependence and Cooperativeness (Ablin et al., 2016; Brooks, Eley & Zink, 2014; Zohar & Cloninger, 2011; Eley, Young & Przybeck, 2008; Eley et al., 2013; Goncalves & Cloninger, 2010; Gutierrez-Zotes et al., 2015; Hansenne, Delhez & Cloninger, 2005). Cross-sectional studies have shown that Novelty Seeking tends to decrease with age, (Cloninger, 2003; Trouillet & Gana, 2008) and in general character traits show changes over time. While Self-Directedness and Cooperativeness normally increase with age, Self-Transcendence decreases until middle age and then increases again as people face ultimate situations like suffering and mortality. Temperament is moderately stable throughout life except that Novelty Seeking decreases and Persistence may increase with age (Cloninger, 2003; Josefsson et al., 2013).

### Rational and aims of this study

This study focused on the association between personality and subjective well-being among a general population sample of Australians. The increase in globalisation and ease of communication internationally suggests we are becoming more alike than different. Studies have shown the economic, social and geographical influences on personality and often find consistent differences between countries and cultures (Kajonius & Mac Giolla, 2017). However, as mentioned above, most of these studies measure personality with the Five-Factor Model. Despite the extant literature using the Temperament and Character Inventory in over 30 countries, as well as several studies of specific participant samples in Australia, there are few using an Australian general population. Using an Australian general population sample, we examined the psychometric properties of the Temperament and Character Inventory (TCIR140) and investigated the associations between TCI-traits with both sociodemographic variables and measures of well-being. In addition, we investigated differences in temperament and character between our Australian general population sample and published results of similar studies from other countries.

## Methods

### Study design and setting

The University of Queensland (UQ) Human Research Ethics Committee (HREC) (2013001083) granted ethical approval for the study. [Approval reference number: UQ HREC 2013001083] All participants provided consent documented on the survey. Participants were recruited from 2016-2017 within the general population *via* community newsletters and national organizations *e.g.*, national seniors, toastmasters, sporting organizations and two universities. In each instance, the organization distributed an announcement about the study through online newsletters or regular publications. The announcement contained a short description of the study, an invitation to participate, and a direct link to the survey for more information. The survey preamble contained the ethical clearance declaration, all participant information and the consent statement. There were no conditions for participation. Participants were offered the option to request their temperament and character personality profile by providing their email address on the survey. All data were collected online *via* Survey Monkey^®^.

### Participants

There were 1772 respondents. Data cleaning excluded those cases that did not provide valid data on the TCIR-140 (the main outcome measure). Invalid responses did not answer at least 80% of the items (*n* = 112; 6%), and/or the four validity items (*n* = 150; 8%). If an individual had answered at least 80% of the items within a scale, the missing data was replaced based on the individual’s average responses on other items within the same scale. The final sample comprised 1510 participants with valid data on TCIR-140. The number of respondents with valid data on PANAS and SWLS were *N* = 1424 and *N* = 1419, respectively. Missing data were handled by pairwise inclusion to retain available information for the corresponding analyses.

### Measures

#### Temperament and Character Inventory Revised-140 [TCIR-140]

The TCIR-140 (Cloninger, 1999) is the 140-item version which uses a five-point Likert-type scale indicating degree of agreement to each statement from 1 = Definitely false to 5 = Definitely true. It measures four traits of temperament; Novelty Seeking (NS), Harm Avoidance (HA), Reward Dependence (RD) and Persistence (PS) and three traits of character; Self-Directedness (SD), Cooperativeness (CO) and Self-Transcendence (ST). Each of the seven traits has 20 items except for Self-Transcendence that has 16. There are four validity items throughout the inventory to account for inattention. The 140 items are presented on a five-point Likert scale (1 = absolutely false to 5 = absolutely true). This study used the Likert score means of each individual trait for analysis and interpretation because the mean scores are easier to interpret and sufficient to our purposes. Interpretation of the TCIR140 trait levels was by mean trait score rankings of the five-point scale as follows: very low (1.00–1.50), low (1.51–2.50), average (2.51–3.50), high (3.51–4.50) and very high (4.51–5.00). File S1 provides a table of descriptors for high and low levels of each trait. The TCIR-140 has been validated widely (Goncalves & Cloninger, 2010; Cloninger & Zohar, 2011; Gutierrez-Zotes et al., 2015).

#### Satisfaction with Life Scale [SWLS]

The SWLS is a measure of subjective well-being (Pavot et al., 1991). It was developed as a five-item scale to assess an individual’s broad judgement of personal life satisfaction. It is administered as a self-report agreement with each item on a seven-point Likert-type scale from 1 = Extremely dissatisfied to 7 = Extremely satisfied and provides a global score. The SWLs is scored by summing the scores on all five items with a higher score indicating a higher satisfaction with life. The SWLS has been validated broadly across many cultures and languages (Goncalves & Cloninger, 2010; Cloninger & Zohar, 2011).

#### The Positive and Negative Affect Scale [PANAS]

The PANAS consists of 20 words that describe different feelings and emotions (Watson, Clark & Tellegen, 1988). A five-point Likert-type scale is provided for individuals to self-report the extent to which they generally feel each item listed on a scale from 1 = Very slightly or not at all to 5 = Extremely. Half of the items describe positive affective states and half negative. The PANAS is scored by adding all the positive affect items together. Higher scores on items and overall indicate higher levels of positive affect. Negative affect is scored similarly by summing all negative items with lower scores on items and overall indicating lower levels of negative affect. The psychometric properties of PANAS have been validated in several cultures (Gyollai et al., 2011).

### Statistical analysis

We examined the descriptive statistics of the TCIR-140, PANAS and SWLS. To assess the reliability of the TCIR-140 descriptive statistics were estimated with Cronbach’s alphas to determine internal consistency.

Confirmatory factor analyses (CFA) were conducted to test the fit of each item into the Temperament and Character models of the TCIR-140. Our data meets the assumptions of a CFA based on assessments of multivariate normality, a sample size of *n* > 300, *a-priori* model specification, and sampling methods. The model structures were pre-determined based on the design of the TCI with four temperament traits in one model, and three character traits in another model. Paths were entered between the latent constructs of the traits based on known significant correlations. These included NS and RD, NS and HA, HA and PS, and HA and RD in the Temperament model, and SD and CO, SD and ST, and CO and ST in the Character model. Better model fit was determined by higher values of comparative fit index (CFI), normed fit index (NFI), Tucker-Lewis coefficient (TLI), and lower values of root mean square error of approximation (RMSEA).

Modification indices were used to inform model building for better model fit using a step-wise approach. Additional paths between the items were put into the model using the step-wise approach based on paths for model fit improvements with the highest values for the purpose of informing the model fit. The results for the a-priori paths of interests were presented because they were pre-determined and relevant to how each item loads onto the construct. The models were specified for paths from the latent dimensions to each observed item. Covariance between the latent subscales were informed by significant correlations between the TCI dimension scores. The CFA and the covariances to be analyzed were conducted using the maximum likelihood method.

The TCIR-140, PANAS and SWLS trait scores were compared by sex and age group using independent sample t-tests and ANOVAs with Bonferroni post hoc correction tests respectively. Partial correlation analyzes, controlling for sex and age, were conducted to examine the association between the TCIR-140 traits with the PANAS and the SWLS.

Lastly, we compared our TCIR-140 trait scores with those from studies of other populations using the same measures. The original data from those studies was not available, therefore, separate one sample t-tests were used to compare our findings with each of the published estimates from different populations. These included samples from an Israel community (Zohar & Cloninger, 2011), a Spanish community (Gutierrez-Zotes et al., 2015) and a Brazilian general population sample (Goncalves & Cloninger, 2010). All analyzes were conducted using SPSS. A significance level of *p* < 0.05 was used in this study.

## Results

### Demographics

Table 1 presents the sample’s demographic characteristics. Females made up 78% of the sample. Age (mean = 44.8 years) ranged from 19 to 90 years and collapsed into three groups, young-adult 19-30 years, mid-adult 31-55 years, and older-adult 56-90 years.

**Table 1:** Demographic characteristics of sample (*N* = 1,510).

	**Frequency**	**Valid %**
**Sex**		
Male	332	22.1
Female	1173	77.9
Missing	5	–
**Age** (mean = 44.84 years; SD = 18.96)		
Young-adult: 19–30	487	32.5
Mid-adult: 31–55	439	29.3
Older-adult: 56–90	573	38.2
Missing	11	–
Relationship status		
Partnered	929	61.9
Single	571	38.1
Missing	10	–
Lived longest		
Urban/Metro	1007	67.5
Regional/Rural	484	32.5
Missing	19	–
**Education level (highest)**		
Postgraduate	411	27.3
Undergraduate	581	38.6
Diploma/Graduate Certificate	230	15.3
Secondary	285	18.9
Missing	3	–

The majority of the sample were partnered, lived longest in an urban/metro location, and lived the majority of their life in Australia. An undergraduate education comprised 38% of the sample. Overall, the participants reflected the demographic characteristics of the Australian general population as described by the Australian Bureau of Statistics report on *People and Communities* (Australian Bureau of Statistics, 2021). As noted above, our sample was recruited from a broad variety of community organizations on a national scale.

### Descriptive statistics and psychometrics

Descriptive statistics of the TCIR-140 traits showed that the sample as a whole scored high (*i.e.,* mean Likert values above 3.5) in levels of Cooperativeness, Self-Directedness and Persistence and average (*i.e.,* mean Likert values of 2.51–3.5) on all other temperament and character traits (see Table 2). There was no strong skewness (skewness ranged from −.66 to .18), in addition, and due to our large sample size and the central limit theorem, the normally assumption of the data were met. Reliability scores (Cronbach alpha) in this sample ranged from *α* = 0.78 for Novelty Seeking to *α* = 0.92 for Harm Avoidance. For the PANAS, high levels of Positive affect (*i.e.,* mean Likert value of 3.5) were found. The whole sample had low levels of Negative affect, with scores skewed to the right (*i.e.,* more participants reporting lower values). The PANAS showed strong reliability in both the Positive affect (*α* = 0.88) and Negative affect (*α* = 0.89) traits. SWLS reliability in this sample was also strong (*α* = 0.88), with more participants having moderately high scores overall, but the skew was not strong (skewness −.73).

**Table 2:** Whole sample descriptive statistics and internal reliability of the Temperament and Character Inventory (TCIR-140), the Positive and Negative Affect Scale and the Satisfaction with Life Scale.

	**Number of items in each scale**	**Cronbach’s alpha (*α*)**	**Mean**	**SD**	**Min**	**Max**
**TCIR-140** ^a^ **(*N* = 1,510)**						
Novelty Seeking	20	.78	2.79	.47	1.40	4.80
Harm Avoidance	20	.92	2.86	.72	1.10	5.00
Reward Dependence	20	.84	3.32	.53	1.20	4.60
Persistence	20	.90	3.55	.56	1.30	4.95
Self-Directedness	20	.90	3.64	.60	1.80	4.90
Cooperativeness	20	.83	3.92	.45	1.75	4.95
Self-Transcendence	16	.89	2.74	.73	1.13	4.75
**Positive & Negative Affect Scale** ^b^ **(*N* = 1,424)**						
Positive Affect	10	.88	35.30	6.47	12.00	50.00
Negative Affect	10	.89	18.29	6.55	10.00	50.00
**Satisfaction with Life Scale** ^c^ **(*N* = 1,419)**	5	.88	24.56	6.51	5.00	35.00

**Notes.**

a Mean TCIR-140 trait scores were measured on a Likert scale of 1–5: Very Low = 1–1.50; Low = 1.52–2.50; Average = 2.51–3.50; High = 3.51–4.50; Very High = 4.51–5.00.

b A separate aggregate score for Positive and Negative Affect is derived from the sum of responses to each item using a five-point Likert scale which is 1 = Very slightly or not at all to 5 = Extremely.

c The sum of responses to a five-point Likert scale from 1 = Extremely dissatisfied to 7 = Extremely dissatisfied, provides a single score for the Satisfaction with Life Scale.

**Figure 1:**
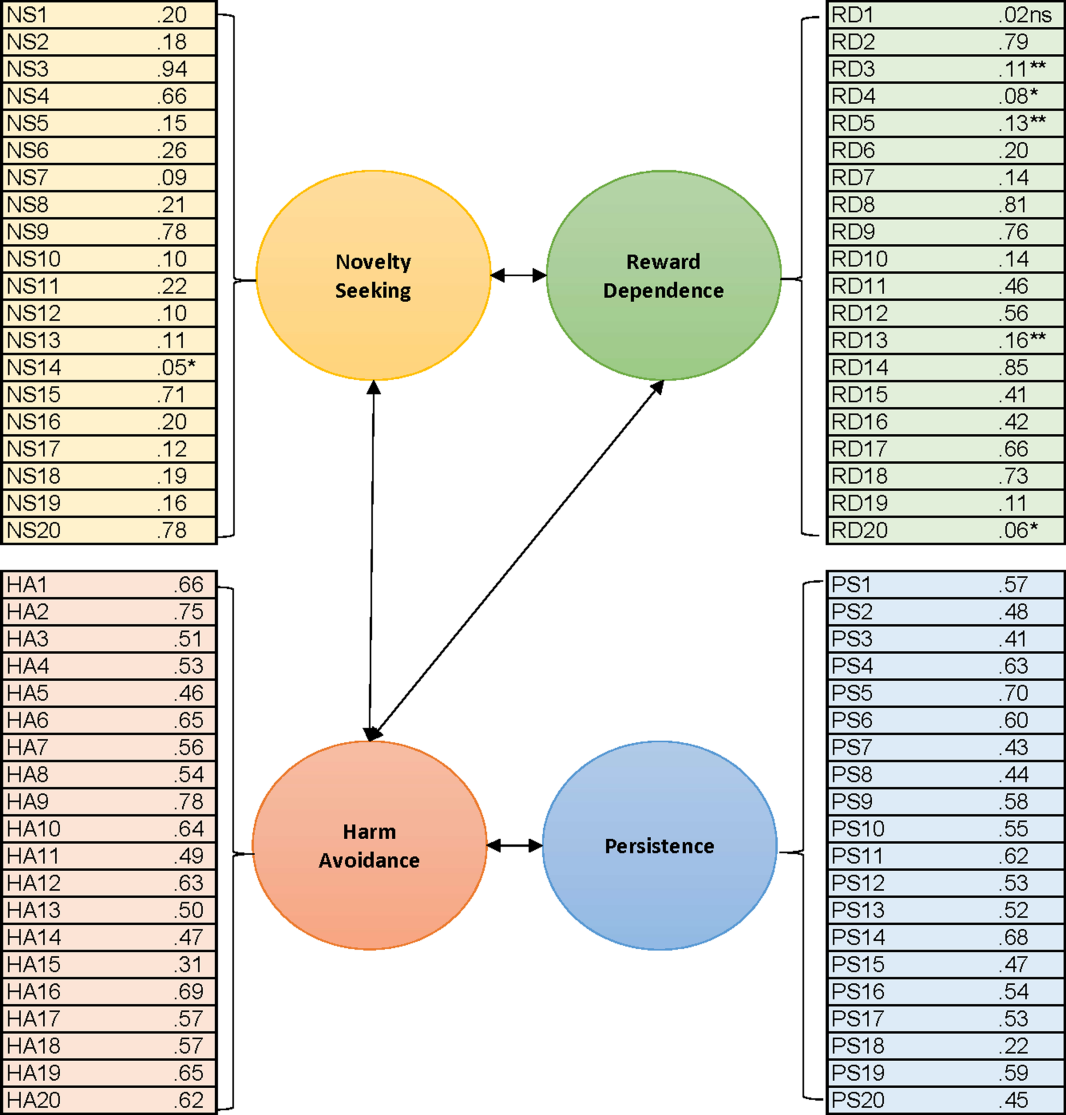
Standardised estimates of the factor loading for each item onto their corresponding construct from the CFA for Temperament subscales. * *p* < 0.05, ** *p* < 0.01, ns—not significant, all others *p* < 0.001.

CFA results for the Temperament dimensions of the TCIR-140 are presented in Fig. 1. Overall, the items loaded moderately well onto their corresponding dimensions, and the model achieved acceptable model fit, CFI = 0.89, NFI = 0.84, TLI = 0.87, RMSEA = 0.039, chi-square = 8182.58, *p* < 0.001. In general, estimates were consistently stronger in the Harm Avoidance and Persistence dimensions, with more variability in Novelty Seeking and Reward Dependence. Only one item did not significantly load onto its designated dimension, but its removal did not significantly improve internal consistency.

**Figure 2:**
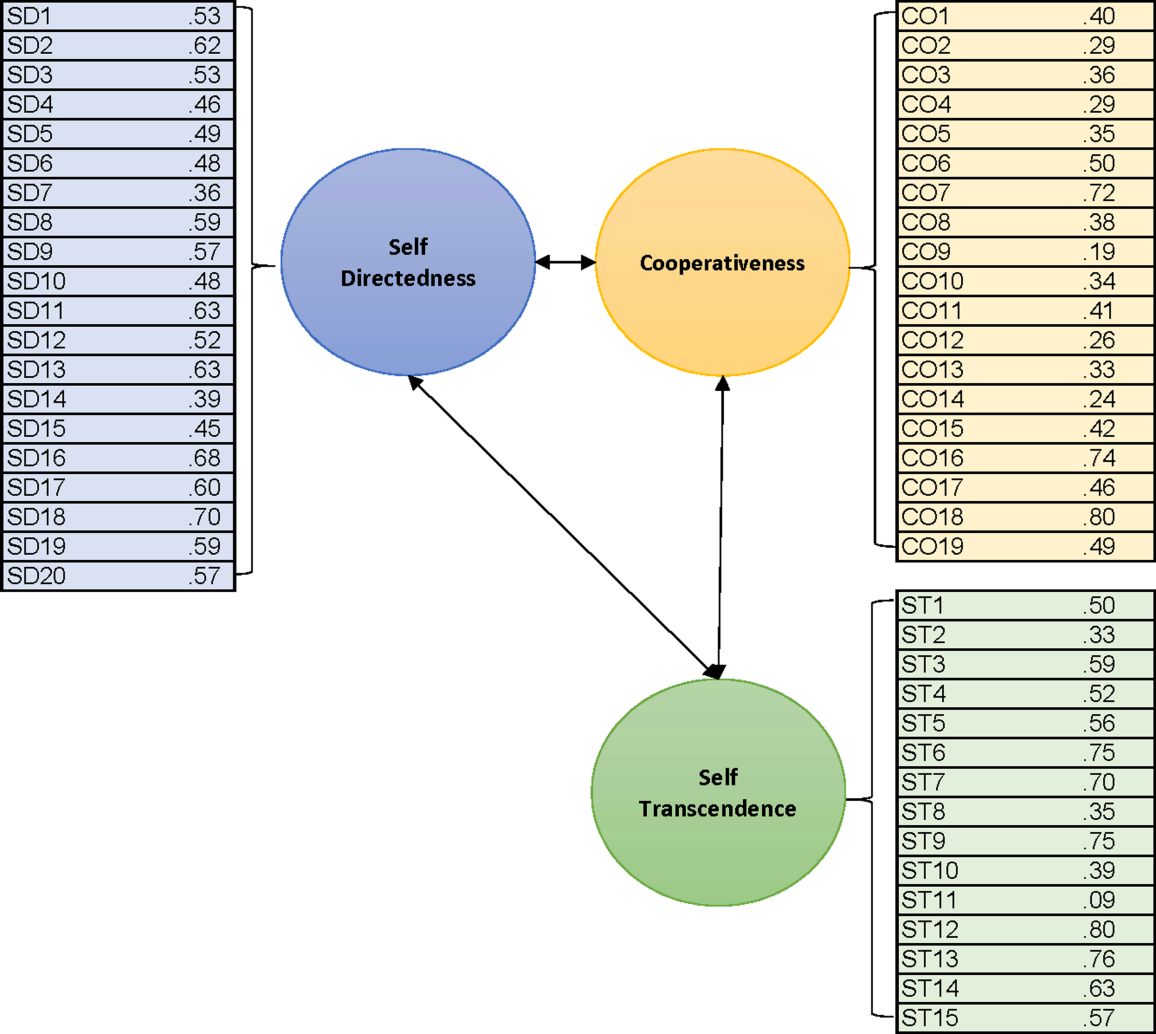
Standardised estimates of the factor loading for each item onto their corresponding construct from the CFA for the Character subscales. *All estimates were statistically significant at *p* < 0.001.

CFA results for the Character dimensions are presented in Fig. 2. Similar to the Temperament model, the Character model results showed that overall, the items loaded moderately onto their corresponding scales, and the model achieved acceptable model fit, CFI = 0.88, NFI = 0.84, TLI = 0.87, RMSEA = 0.041, chi-square = 4298.53, *p* < 0.001. One item with the lowest standardised estimate loaded weakly on its scale, but its removal would only improve internal consistency by 0.01.

### Correlation between the TCIR-140 with PANAS and SWLS

Table 3 provides partial correlations among the TCIR-140 traits PANAS and SWLS, controlling for sex. Significant moderate to strong correlations (*r* > 0.3) were found between Harm Avoidance with Persistence (*r* =  − 0.36, *p* < 0.001) and Self-Directedness (*r* =  − 0.61, *p* < 0.001), and between Cooperativeness with Reward Dependence (*r* = 0.44, *p* < 0.001), and Self-Directedness (*r* = 0.40, *p* < 0.001). Greater Positive Affect scores were correlated significantly with lower Harm Avoidance (*r* =  − 0.57, *p* < 0.001), higher Persistence (*r* = 0.54, *p* < 0.001) and higher Self-Directedness (*r* = 0.54, *p* < 0.001). Greater Negative Affect scores were correlated with higher Harm Avoidance (*r* = 0.56, *p* < 0.001) and lower Self-Directedness (*r* =  − 0.60, *p* < 0.001). Greater SWLS scores were correlated with lower Harm Avoidance (*r* =  − 0.38, *p* < 0.001) and higher Self-Directedness (*r* = 0.58, *p* < 0.001). Because the majority of the sample is female Appendix 2 shows individual correlations by male and by female. Here we see the strength of several correlations greater in males than females. See File S2 for more detail.

**Table 3:** Partial correlations of the Temperament and Character Inventory (TCIR-140), Positive and Negative Affect Scale (PANAS) and Satisfaction with Life Scale (SWLS) controlling for sex (*N* = 1,505).

	**NS**	**HA**	**RD**	**PS**	**SD**	**CO**	**ST**	**PA**	**NA**	**SWL**
Novelty Seeking (NS)	–									
Harm Avoidance (HA)	0.21	–								
Reward Dependence (RD)	0.15	−0.15	–							
Persistence (PS)	−0.05	−0.36^*^	0.04	–						
Self-Directedness (SD)	−0.17	−0.61^*^ ^a^	0.15	0.39^*^	–					
Cooperativeness (CO)	−0.08	−0.28^*^	0.44^*^	0.16	0.40^*^	–				
Self-Transcendence (ST)	0.13	−0.19^*^	0.21^*^	0.18	0.06	0.28^*^	–			
Positive Affect (PA)	0.06	−0.57^*^	0.23^*^	0.54^*^	**0.54** ^*^	0.26^*^	0.28^*^	–		
Negative Affect (NA)	0.06	0.56^*^	−0.10	−0.16	**−0.60** ^*^	**−0.30** ^*^	0.07	−0.23^*^	–	
Satisfaction with Life (SWL)	0.09	−0.38^*^	0.14	0.24^*^	**0.58** ^*^	0.22	0.12	0.44^*^	−0.38^*^	–

**Notes.**

* *p* < 0.001.

a Correlations greater than 0.3 are indicated in bold font.

### Association of demographics with the TCIR-140, PANAS and SWLS

The mean scores on the 5-point Likert scale for the TCIR-140 centred near three and the whole sample scored high (above 3.5) in levels of Persistence, Cooperativeness and Self-Directedness. Scores were average on all other traits. Comparison of variable mean scores for TCIR-140, PANAS and SWLS by sex and age are presented in Table 4.

**Table 4:** Likert mean scores and standard deviations (SD) for the Temperament and Character Inventory, Positive and Negative Affect Scale and Satisfaction with Life Scale, and comparisons by sex and age.

**Table 4a**	**Comparisons by SEX**				
	Male: *n* = 332		Female: *n* = 1,173		*p*
**TCIR-140** ^a^	Mean	(SD)	Mean	(SD)	
Novelty Seeking	2.75	(0.43)	2.80	(0.48)	0.12
Harm Avoidance	2.64	(0.63)	**2.93**	(0.73)	<.001
Reward Dependence	3.21	(0.52)	**3.35**	(0.53)	<.001
Persistence	3.56	(0.56)	3.55	(0.55)	0.74
Self-Directedness	**3.71**	(0.59)	3.62	(0.61)	<0.01
Cooperativeness	3.81	(0.45)	**3.95**	(0.45)	<.001
Self-Transcendence	2.70	(0.76)	2.75	(0.72)	0.24
	Male: *n* = 316		Female: *n* = 1,099		*p*
**PANAS** ^b^					
Positive Affect	35.52	(6.92)	35.24	(6.34)	0.48
Negative Affect	17.76	(5.97)	18.43	(6.67)	0.11
**Satisfaction with Life Scale** ^c^	24.87	(6.02)	24.48	(6.65)	0.34

**Table 4b**	**Comparisons by AGE**						
	19–30 (*n* = 487) **Young adult**		31–55 (*n* = 439) Mid adult		56–90 (*n* = 573) Older adult		*p*
**TCIR-140**$	Mean	(SD)	Mean	(SD)	Mean	(SD)	
Novelty Seeking	2.85	(0.51)	2.84	(0.48)	**2.69** ^d^	(0.40)	<.001
Harm Avoidance	**3.17** ^d^	(0.79)	2.75	(0.67)	2.71	(0.60)	<.001
Reward Dependence	3.31	(0.57)	3.31	(0.54)	3.33	(0.49)	0.69
Persistence	3.50	(0.63)	**3.64** ^d^	(0.53)	3.53	(0.50)	<.001
Self-Directedness	**3.40** ^d^	(0.62)	3.72	(0.57)	3.77	(0.55)	<.001
Cooperativeness	**3.76** ^d^	(0.50)	3.99	(0.44)	4.00	(0.39)	<.001
Self-Transcendence	**2.59** ^d^	(0.72)	**2.72** ^d^	(0.72)	**2.89** ^d^	(0.71)	<.001
	**Young adult**		**Mid adult**		**Older adult**		*p*
**PANAS** ^b^	*n* = 431		*n* = 416		*n* = 567		
Positive Affect	**33.33** ^d^	(6.81)	35.76	(6.47)	36.39	(5.93)	<.001
Negative Affect	**21.01** ^d^	(7.05)	17.42	(5.90)	16.91	(5.93)	<.001
**Satisfaction with Life Scale** ^c^	**23.99** ^d^	(6.70)	25.01	(6.56)	24.63	(6.27)	<0.01

**Notes.**

a Mean TCIR-140 trait scores were measured on a Likert scale of 1–5: Very Low = 1–1.50; Low = 1.52–2.50; Average = 2.51–3.50; High = 3.51–4.50; Very High = 4.51–5.00.

b A separate aggregate score for Positive and Negative Affect is derived from the sum of responses to each item using a five point Likert scale which is 1 = Very slightly or not at all to 5 = Extremely.

c The sum of responses to a five point Likert scale from 1 = Extremely dissatisfied to 7 = Extremely dissatisfied, provides a single score.

d Bold type indicates which variable is significantly different from the others using Bonferoni Post Hoc tests.

#### Sex.

Table 4a shows that females had significantly higher levels of Harm Avoidance (female *M* = 2.93 [SD = 0.73], male *M* = 2.64 [0.63], *p* < 0.001), Reward Dependence (female *M* = 3.35 [0.53] male *M* = 3.21 [0.52], *p* < 0.001), and Cooperativeness (female *M* = 3.95 [0.45], male *M* = 3.81 [0.45], *p* < 0.001). Males had significantly higher levels of Self-Directedness (female *M* = 3.62 [0.61], male *M* = 3.71 [0.59], *p* < 0.001). No differences were found between males and females in the PANAS or SWLS.

#### Age.

Table 4b compares the TCIR-140 between age groups and showed significant differences among all traits (*p* < 0.001) except for Reward Dependence. Bonferroni post hoc tests showed that for the Temperament traits, the older-adults were lowest in Novelty Seeking (means [SD] in the young, mid-aged, and older adult groups were: *Y* = 2.85 [0.51], *M* = 2.84 [0.48], *O* = 2.69 [0.40], *p* < 0.001). For Harm Avoidance, the young-adults had the highest levels (*Y* = 3.17 [0.79], *M* = 2.75 [0.67], *O* = 2.71 [0.60], *p* < 0.001), and mid-adults were highest in Persistence (*Y* = 3.50 [0.63], *M* = 3.64 [0.53], *O* = 3.53 [0.50], *p* < 0.001). For the Character traits, young-adults were lowest in Self-Directedness (*Y* = 3.40 [0.62], *M* = 3.72 [0.57], *O* = 3.77 [0.55], *p* < 0.001) and Cooperativeness (*Y* = 3.76 [0.50], *M* = 3.99 [0.44], *O* = 4.00 [0.39], *p* < 0.001). Self-Transcendence among all three age groups were significantly different from each other, increasing with age (*Y* = 2.59 [0.72], *M* = 2.72 [0.72], *O* = 2.89 [0.71], *p* < 0.001). Comparing the PANAS by age shows young-adults as lower in Positive Affect (*Y* = 33.33 [6.81], *M* = 35.76 [6.47], *O* = 36.39 [5.93], *p* < 0.001) and higher in Negative Affect (*Y* = 21.01 [7.05], *M* = 17.42 [5.90], *O* = 16.91 [5.93], *p* < 0.001). The young-adults are also lower in satisfaction with life compared to the older groups (*Y* = 23.99 [6.70], *M* = 25.01 [6.56], *O* = 24.63 [6.27], *p* < 0.01).

#### Relationship status.

There were several differences in the temperament and character and well-being depending on marital status. Respondents who were single scored higher on Harm Avoidance [single = 2.92 [0.74], married/partnered = 2.83 [0.71], *t* =  − 2.36; *df* = 1498, *p* = 0.02], lower on Self-Directedness [single = 3.56 [0.62], married/partnered = 3.69 [0.59], *t* = 4.09; *df* = 1498, *p* < 0.001], higher on Negative Affect [ *t* =  − 2.76; *df* = 1498, *p* = 0.001], and lower on Satisfaction with Life [ *t* = 5.344; *df* = 1498, *p* < 0.001] compared to partnered respondents.

#### Education.

Comparing the traits by the highest level of education used a Bonferonni post hoc test to show differences within the multiple comparisons. Respondents with a postgraduate degree showed highest levels of Reward Dependence [postgraduate = 3.38 [0.52], secondary = 3.34 [0.53], *F* = 3.31; (3) *p* = 0.02], Self-Directedness [postgraduate = 3.74 [0.57], secondary = 3.51 [0.62], *F* = 10.73; (3) *p* < 0.001], and Satisfaction with Life [ *F* = 9.17; (3) *p* < 0.001 < 0.001]. Those reporting a secondary education were highest in levels of Harm Avoidance [postgraduate = 2.80 [0.70], secondary = 2.97 [0.73], *F* = 3.32; (3) *p* = 0.02], lowest in Persistence [postgraduate = 3.63 [0.54], secondary = 3.45 [0.58], *F* = 6.02; (3) *p* < 0.001 < 0.001], and highest in Negative Affect [ *F* = 5.70; (3) *p* = 0.001].

#### Location Lived longest.

No differences were detected between respondents who reported living longest in an urban-metro compared to a rural-regional location.

### Comparisons with other countries and cultures

The mean TCIR-140 trait scores from this study were compared with scores from similar general population/community studies and are presented in Table 5. Brief demographics of the comparator country samples are also listed on Table 5. Compared to the Spanish sample, Australians are significantly higher in Novelty Seeking, Persistence and Self-Transcendence and lower in every other trait. The Australians differ from the Israeli sample being significantly higher in Harm Avoidance, Persistence, Cooperativeness and Self-Transcendence. Compared to Brazilians, our sample is higher in Harm Avoidance and Cooperativeness, and lower in Reward Dependence and Self-Transcendence.

**Table 5:** Comparison of Australian mean levels and standard deviations (SD) of the Temperament and Character (TCIR-140) traits with those of other general population samples.

**TCIR-140**	Australian general population *N* = 1,505	Israeli community^a^*N* = 1,102	*p*	Spanish community^b^*N* = 367	*p*	Brazilian community^c^*N* = 595	*p*
	Mean (SD)	Mean (SD)		Mean (SD)		Mean (SD)	
Novelty Seeking	**2.79 (0.47)**	2.77 (0.35)	0.180	**2.54 (0.47)** ^d^	**<.001**	2.81 (0.38)	0.047
Harm Avoidance	**2.87 (0.72)**	**2.64 (0.55)** ^d^	**<.001**	**3.02 (0.58)** ^d^	**<.001**	**2.71 (0.23)** ^d^	**<.001**
Reward Dependence	**3.32 (0.53)**	3.32 (0.45)	0.999	**3.56 (0.54)** ^d^	**<.001**	**3.46 (0.46)** ^d^	**<.001**
Persistence	**3.55 (0.56)**	**3.31 (0.46)** ^d^	**<.001**	**3.19 (0.64)** ^d^	**<.001**	3.56 (0.50)	0.557
Self-Directedness	**3.63 (0.55)**	3.64 (0.54)	0.821	**3.84 (0.61)** ^d^	**<.001**	3.64 (0.48)	0.821
Cooperativeness	**3.92 (0.45)**	**3.80 (0.40)** ^d^	**<.001**	**4.03 (0.48)** ^d^	**<.001**	**3.86 (0.39)** ^d^	**<.001**
Self-Transcendence	**2.74 (0.73)**	**2.52 (0.67)** ^d^	**<.001**	**2.37 (0.70)** ^d^	**<.001**	**2.97 (0.53)** ^d^	**<.001**
Brief demographics							
Sex (% male)	22	37	–	43	–	40	–
Mean Age (years)	45	58	–	42	–	33	–
Partnered (%)	62	69	–	64	–	N/A	–
Education (majority)	Undergraduate & Secondary	Undergraduate & Secondary	–	Secondary	–	Secondary	–

**Notes.**

a Zohar & Cloninger (2011).

b Gutierrez-Zotes et al. (2015).

c Goncalves & Cloninger (2010).

d Bold type indicates which variable is significantly different from the Australian sample. Original data was not available and therefore one-sample *t*-tests were used.

## Discussion

This study provides a description of temperament and character among a sample of the general population in Australia and the relationship with measures of well-being, and a comparison with similar studies in other countries. The psychometric properties and validity of the TCIR-140 in this Australian sample was also explored. The psychometric properties of the TCIR-140 proved to be satisfactory with good internal consistency for all seven dimensions. Our confirmatory analysis supported the four-subscale Temperament model and the three-subscale Character model. The following will discuss the main findings of the study which describe the Australian temperament and character profile and the associations with demographic characteristics and measures of well-being while also making comparisons to the international literature focusing on the three comparator countries in this study.

### Australian temperament and character profile and associations with demographic characteristics

The temperament and character associations with the sample demographics were consistent with previous literature that shows females are higher in levels of Harm Avoidance, Reward Dependence and Cooperativeness compared to males (Hansenne, Delhez & Cloninger, 2005; Preiss et al., 2007; Goncalves & Cloninger, 2010; Cloninger & Zohar, 2011; Gutierrez-Zotes et al., 2015). Men were higher than females only in Self-Directedness but levels were not different in Persistence. This is contrary to studies in Israel and Belgium which found men to be higher than women in both traits (Zohar & Cloninger, 2011; Hansenne, Delhez & Cloninger, 2005), and with a French study showing men to be higher in Reward Dependence, Cooperativeness and Self-Directedness (Pelissolo et al., 2005). Schmitt et al. (2017) has proposed that counties with greater egalitarian cultures show greater differences in psychological constructs by gender. Australia is considered a democratic society yet differences in certain traits, common in other societies are not seen. A suggestion might be related to the gender-equality paradox which has been observed in relation to females entering STEM (science technology engineering and maths) degrees (Fors, Goossen & Hjerm, 2020). Nevertheless in keeping with the literature, significantly higher Harm Avoidance in females is an almost universal finding (Mendlowicz & Girardin, 2000; Miettunen et al., 2006), and is likely to be related to levels of anxiety shown to be more prevalent among females (Cloninger, 2006).

Our findings related to age are also largely consistent with the international literature using the TCIR-140. Novelty Seeking decreased with age (Aluja et al., 2010; Cloninger & Zohar, 2011; Fresán et al., 2011; Gutierrez-Zotes et al., 2015; Josefsson et al., 2013; Preiss et al., 2007). However, contrary to Gutierrez-Zotes et al. (2015), Harm Avoidance also decreased with age in this Australian sample. Furthermore, in our sample, levels of Self-Directedness and Cooperativeness were highest in older adults compared to the two younger groups. Reward Dependence was not significant between age groups but levels of Self-Transcendence, although significantly different between each group, were highest in the older adults.

In comparison to partnered respondents, those who were single scored higher on Harm Avoidance and lower on Self-Directedness, which fit with lower scores on SWL and higher Negative Affect. Partnered individuals may feel more settled and fulfilled with a steady partner and therefore are less anxious and more positive for their future. Respondents with a postgraduate degree had the highest levels of Self-Directedness and Reward Dependence, which suggests they are conscientious and highly sociable which may be characteristics valuable to earning a higher education degree. In contrast those with a secondary education had the lowest levels of Persistence and highest levels of Harm Avoidance and Negative Affect.

### Temperament and character associations with measures of well-being

A unique aspect of our study is the inclusion of life satisfaction, and positive and negative affect, as proxy measures of subjective well-being, and to measure their associations with temperament and character traits. Correlations were calculated between all TCIR-140 traits with the PANAS and SWLS. Harm Avoidance and Self-Directedness had a contrasting pattern of association with all the others. Harm Avoidance was positively correlated with Negative Affect, and negatively to Positive Affect and SWLS. The opposite was seen with Self-Directedness, negatively correlated with Negative Affect but positive with Positive Affect and SWLS. Only Persistence was also strongly correlated with Positive Affect, but all other associations were weak. Zohar & Cloninger (2011) showed this same pattern in their Israeli sample. The pattern of correlations between Harm Avoidance and Persistence show temperament closely related to well-being, as is the character trait Self-Directedness and Cooperativeness. A profile of temperament that is low in levels of Harm Avoidance, and high in Persistence, alongside, a character of high Self-Directedness and Cooperativeness has been shown to be strongly associated with high levels of resilience (Eley et al., 2013; Eley et al., 2017) and well-being (Cloninger & Zohar, 2011; Josefsson et al., 2013).

According to Diener (2009), personality is strongly associated with well-being and there is substantial stability in subjective well-being over time. This is due in part to the heritable dimensions of personality being relatively stable throughout life. Our sample’s mean levels of each temperament and character trait follow this pattern, distinguished by average Harm Avoidance, indicating confidence and the ability to tolerate a level of uncertainty, plus high Persistence demonstrating industriousness and tenacity. This temperament will be relatively stable throughout life and complimented by a character prone to being highly Self Directed, responsible and conscientious, as well as highly Cooperative which speaks to the overall character of Australians.

Therefore, it is the combination and levels of each trait; stable temperament and character influenced by life experience, that helps us perceive our well-being and explain personality over time. In our sample, young adults had the highest levels of Harm Avoidance, and the lowest levels of Persistence. Self-Directedness and Cooperativeness. Essentially the opposite of the profile most conducive to well-being and perhaps unsurprisingly as they also scored lowest on Satisfaction with Life and Positive Affect and highest on Negative Affect. Sadly, this finding may reflect the rising concern over the poor mental health in our young people.

### Comparison with similar temperament and character studies in other countries

Looking at the inter-correlation between the temperament and character traits across studies showed that although the trend of all associations is in the expected direction, this sample showed some moderate to strong relationships that differ from the others. For example, the negative correlation between Harm Avoidance with Persistence and Self-Directedness was higher, and the positive relationship between Cooperativeness with Reward Dependence and Self Directedness was also higher compared to those reported in the Spanish sample by Gutierrez-Zotes et al. (2015).

While an in-depth comparison of the temperament and character trait levels across the three countries was not within the scope of the study, it is of interest to note how differences may relate to the cultural values in each country. According to the data from the World Values Survey, the longitudinal database providing worldwide data on social, cultural and political change (Inglehart & Baker, 2000), the dimension of Traditional/Secular-rational values can be an indicator of a country’s values which is likely to be reflected broadly in the personality of their population. This dimension distinguishes between societies where religion and traditional values are very important and may feature in their laws and societal norms *i.e.,* traditional, and those that are not *i.e.,* secular. Australia is around the halfway point but leaning toward secular values along with much of Europe, including Spain and also Israel, while Brazil is more traditional.

Comparison of TCIR-140 trait levels with similar studies in other populations showed this Australian sample to be significantly different in mean levels of all seven traits to a Spanish sample (Gutierrez-Zotes et al., 2015). The Spanish sample in general is higher in levels of all the temperament and character traits across the three countries. The Australian sample is higher in Harm Avoidance, Persistence, Cooperativeness and Self-Directedness compared to the Israeli study (Zohar & Cloninger, 2011). In comparison to the Brazilian sample (Goncalves & Cloninger, 2010), Australians are higher in Harm Avoidance, and Cooperativeness, and lower in Reward Dependence and Self-Transcendence. When compared with the Brazilian and Israeli samples, Australians in this sample have a cautious and independent nature with a character that is cooperative, industrious, and self-reliant.

## Limitations

This study has some limitations. It was cross-sectional and associational and therefore does not infer causality nor can it determine direction underlying any of our associations. Our findings are limited by the inherent bias that comes with self-reported data and volunteer recruitment. Studies have shown that volunteers for research may differ from the general population in certain personality traits such as agreeableness and extroversion (Dollinger & Leong, 1993; Saliba & Ostojic, 2014). Recruitment for this study displayed the commonly seen bias of an overrepresentation of females (Zohar & Cloninger, 2011; Goncalves & Cloninger, 2010; Gutierrez-Zotes et al., 2015; Vella-Brodrick, Park & Peterson, 2009). For this reason, we presented and compared our findings stratified by participants’sex. Furthermore, our sample was biased towards mid to older adult age groups (Cloninger & Zohar, 2011; Goncalves & Cloninger, 2010; Headey, Muffels & Wagner, 2013). However, we achieved a broad age range that lent itself to stratification into nearly equal groups that are representative of young, middle and older adult stage of life. Our sample had higher levels of education than the general population, which may impact our univariate results on the levels of psychological measures. It is unlikely that this would cause major bias in our results on the relationship between personality, affect, and quality of life because our sample displayed a spread of responses on these measures. An advantage of our large national sample is its broad representation of Australians. However, studies should also look at more regional variations in personality across our vast geography.

Another limitation is that we did not collect race or ethnicity data. This was in an attempt to reduce questions of a private and potentially identifiable nature. However, Australia is very multicultural country and this would have provided another interesting dimension to our findings.

We recognize that an advantage of using the Biopsychosocial Model of Personality is the distinction between the two dimensions of personality and the interaction of both within- and between-person traits. While an exploration of this distinction in personality as profiles or individual traits would have been relevant and important especially when comparing across countries, the additional depth required in design and analysis was beyond our scope (Cloninger & Zwir, 2018). This is a limitation of our study that should be included in future work.

## Conclusions

This study provides new information about the temperament and character of Australians. It validated the Temperament and Character Inventory(TCIR-140) in a large general population sample of Australians and found that the psychometric properties were good. Overall, our sample demonstrates a temperament that is high in Persistence and a character high in Self-Directedness and Cooperativeness. Compared to males, females show a strong and similar pattern of higher Harm Avoidance, Reward Dependence and Cooperativeness, which is consistent with the literature. However, our study found that men are higher than females only in Self-Directedness. Age trends show that young adults score lowest in well-being measures and have a temperament and character profile that describes this, in particular; higher Harm Avoidance and lower Self-Directedness and Cooperativeness compared to the older groups. The majority of the whole sample scored high for positive affect and low for negative affect, with moderately high scores on satisfaction with life. Compared to similar studies in other countries, Australians in this sample demonstrate a cautious and independent temperament with a character that is cooperative, industrious, and self-reliant. Future research should explore in more detail the perception of subjective well-being in young adults. Understanding particular temperament and character profiles may help identify how to strengthen certain traits to build a more resilient personality and positive outlook for their future.

##  Supplemental Information

10.7717/peerj.15342/supp-1 Table S1 High and low descriptors for each temperament and character trait Adapted from Eley et al, 2016.

10.7717/peerj.15342/supp-2 Table S2 Pearson’s correlations among all variables by sex (*N* = 1,505)

10.7717/peerj.15342/supp-3 Data S1 Raw Data

## Additional Information and Declarations

### Data Availability

The following information was supplied regarding data availability: The raw measurements are available in the Supplementary File.
