# Supplementary material for: Temperament and character in an Australian sample: examining cross-sectional associations of personality with age, sex, and satisfaction with life
Source: PeerJ. 2023 May 11;11:e15342. doi: 10.7717/peerj.15342 (PMC10183160; doi:10.7717/peerj.15342)
Supplement: Table S1 — Adapted from Eley et al, 2016. [file peerj-11-15342-s001.docx]

# Supplemental Table 1:

# High and low descriptors for each temperament and character trait. ^†^

| **Temperament traits** | **Represents** | **LOW SCORES** |  | **HIGH SCORES** |
| --- | --- | --- | --- | --- |
| Novelty Seeking | Exploratory activity in response to novelty | Orderly, reflective, reserved | **<--->** | Exploratory, curious, |
|  |  |  |  | seeks challenge |
| Harm Avoidance | Worry in anticipation of problems | Confident, accepting of uncertainty & risk | **<--->** | Anxious, uncomfortable |
|  |  |  |  | with accepting risk |
| Reward Dependence | Dependence on approval of others | Not influenced by others, objective, insensitive | **<--->** | Needs to please, warm, attached, sociable |
| Persistence | Industriousness of behaviour despite obstacles | Quitting, underachiever, erratic, unambitious | **<--->** | Ambitious, hard worker, diligent, |
|  |  |  |  | perfectionist |
| **Character** | **Represents** | **LOW SCORES** | **<--->** | **HIGH SCORES** |
| **traits** |  |  |  |  |
| Self-Directedness | Responsibility, goal orientated & self-confidence | Blaming, ineffective, unreliable, irresponsible | **<--->** | Conscientious, |
|  |  |  |  | self-accepted, |
|  |  |  |  | reliable, |
| Cooperativeness | Tolerance, cooperativeness & empathy | Intolerant, unhelpful opportunistic, | **<--->** | Tolerant, agreeable, constructive, |
|  |  | critical |  | empathic |
| Self-Transcendence | View of self in relation to the universe as a whole | Impatient, proud, materialistic, practical | **<--->** | Patient, humble, |
|  |  |  |  | spiritual, creative, compassionate |
| Adapted from Eley et al, 2016 ^†^ | | | | |
