# Supplementary material for: Temperament and character in an Australian sample: examining cross-sectional associations of personality with age, sex, and satisfaction with life
Source: PeerJ. 2023 May 11;11:e15342. doi: 10.7717/peerj.15342 (PMC10183160; doi:10.7717/peerj.15342)
Supplement: Table S2 [file peerj-11-15342-s002.docx]

# Supplemental Table 2: Pearson's correlations among all variables by sex (N=1505).

|  | **NS**† | **HA** | **RD** | **PS** | **SD** | **CO** | **ST** | **PA** | **NA** |
| --- | --- | --- | --- | --- | --- | --- | --- | --- | --- |
| **Male (N = 332)** | | | | | | | |  |  |
| Novelty Seeking | – |  |  |  |  |  |  |  |  |
| Harm Avoidance | -0.24*** | - |  |  |  |  |  |  |  |
| Reward Dependence | 0.21*** | **-0.31***** | - |  |  |  |  |  |  |
| Persistence | 0.02 | **-0.42***** | 0.04 | - |  |  |  |  |  |
| Self-Directedness | -0.18** | **-0.65***** | 0.18** | **0.43***** | - |  |  |  |  |
| Cooperativeness | -0.09 | **-0.30***** | **0.50***** | 0.05 | **0.37***** | - |  |  |  |
| Self-Transcendence | 0.18****** | **-0.35***** | 0.28*** | 0.23*** | 0.18** | **0.30***** | – |  |  |
| Positive Affect | 0.03 | **-0.61***** | 0.25** | **0.65***** | **0.62***** | 0.21*** | **0.32***** | - |  |
| Negative Affect | 0.04 | **0.47***** | -0.12* | -0.12 | **-0.48***** | -0.28** | -0.08* | -0.12 | - |
| Life Satisfaction | -0.12* | **-0.45**** | 0.17* | 0.25** | **0.59***** | 0.26*** | 0.27*** | **0.45***** | **-0.39***** |
| **Female (N = 1173)** | | | | | | | |  |  |
| Novelty seeking | – |  |  |  |  |  |  |  |  |
| Harm avoidance | -0.21*** | - |  |  |  |  |  |  |  |
| Reward dependence | 0.12*** | -0.14*** | - |  |  |  |  |  |  |
| Persistence | -0.07* | **-0.35***** | 0.04 | - |  |  |  |  |  |
| Self-directedness | -0.17*** | **-0.59***** | 0.15*** | **0.37***** | - |  |  |  |  |
| Cooperativeness | -0.09** | **-0.31***** | **0.41***** | 0.20*** | **0.42***** | - |  |  |  |
| Self-Transcendence | 0.12*** | -0.15*** | 0.19*** | 0.16*** | 0.03 | 0.28*** | – |  |  |
| Positive Affect | 0.07* | **-0.57***** | 0.22*** | **0.51***** | **0.51***** | 0.28*** | 0.26*** | - |  |
| Negative Affect | 0.07* | **0.58***** | -0.09** | -0.18*** | **-0.63***** | **-0.31***** | 0.28 | -0.27*** | - |
| Life Satisfaction | -0.09* | **-0.36***** | 0.13** | 0.23*** | **0.57***** | 0.21*** | 0.08** | **0.44***** | -**0.38***** |

† NS = Novelty Seeking; HA = Harm Avoidance; RD = Reward Dependence; PS = Persistence; SD = Self-Directedness CO = Cooperativeness; ST = Self-Transcendence; PA= Positive Affect; NA = Negative Affect; SWL = Satisfaction with Life

*p < .05, ** p < .01, *** p < .001; n = 4 with missing data on sex; Correlations greater than 0.3 are indicated in **bold** font.
